# Luminal Breast Cancer Cell Lines Overexpressing ZNF703 Are Resistant to Tamoxifen through Activation of Akt/mTOR Signaling

**DOI:** 10.1371/journal.pone.0072053

**Published:** 2013-08-26

**Authors:** Xi Zhang, Xin Mu, Ou Huang, Zuoquan Xie, Min Jiang, Meiyu Geng, Kunwei Shen

**Affiliations:** 1 Comprehensive Breast Health Center, Ruijin Hospital, Shanghai Jiaotong University School of Medicine, Shanghai, China; 2 Department of Urology, Renji Hospital, Shanghai Jiaotong University School of Medicine, Shanghai, China; 3 Division of Antitumor Pharmacology, State Key Laboratory of Drug Research, Shanghai Institute of Materia Medica, Chinese Academy of Sciences, Shanghai, China; H.Lee Moffitt Cancer Center & Research Institute, United States of America

## Abstract

**Background:**

Selective estrogen receptor modulators, such as tamoxifen, play a pivotal role in the treatment of luminal-type breast cancer. However, in clinical applications, nearly half of breast cancer patients are insensitive to tamoxifen, a small number of whom have early recurrence or disease progression when receiving tamoxifen. The underlying mechanism of this resistance has not been determined. ZNF703 is a novel oncogene in the 15% of breast cancers that harbor 8p12 amplifications. Therefore, the goal of our study was to explore the role of ZNF703 in tamoxifen resistance.

**Methodology/Principal Findings:**

We used immunohistochemistry techniques to examine ZNF703 expression in stage I-III primary breast cancer specimens and found a positive expression rate of 91.3%. All patients were divided into either high or low ZNF703 expression groups. We found that high ZNF703 expression mainly occurred in ER+ and PR+ breast cancers. Furthermore, 4-hydroxytamoxifen had different modes of action in breast cancer cell lines with high or low ZNF703 expression. ZNF703 overexpression in MCF-7 breast cancer cells activated the Akt/mTOR signaling pathway, downregulated ERα, and reduced the antitumor effect of tamoxifen. Low-dose tamoxifen did not suppress, but rather, stimulated the growth of cells overexpressing ZNF703. ZNF703 knockdown in MDA-MB-134 and HCC1500 luminal B-type breast cancer cell lines by siRNA significantly decreased survival rates when cells were treated with tamoxifen. Furthermore, targeting ZNF703 with a mTOR inhibitor increased the inhibitory effects of tamoxifen in ZNF703-overexpressing cells.

**Conclusion/Significance:**

Our study suggests that ZNF703 expression levels may predict tamoxifen sensitivity. Tamoxifen should be administered with caution to those patients bearing tumors with ZNF703 overexpression. However, large clinical trials and prospective clinical studies are needed to verify these results.

## Introduction

The molecular typing of breast cancer provides a basis for the prognosis and treatment of breast cancer. There are four major molecular subtypes of breast cancers: triple negative/basal-like, human epidermal growth receptor (HER)-2 positive, Luminal A, and Luminal B [1]. Most breast cancers are luminal tumors. Luminal A and B tumors tend to be estrogen receptor-positive (ER+) and/or progesterone receptor-positive (PR+). A variety of endocrine therapies act through different mechanisms to antagonize the growth of tumors stimulated by estrogen. Selective estrogen receptor modulators (SERM), such as tamoxifen, can antagonize ERα activity, and have been used in breast cancer therapy. Tamoxifen is very effective for the treatment of luminal breast cancer; 5 years of tamoxifen therapy can reduce the risk of recurrence and death by 41% and 33%, respectively [2]. However, of those patients who receive adjuvant tamoxifen therapy for 5 years, 8% have early recurrence (within less than 2.5 years), and another 8% have recurrence within 2.5 years to 5 years [3]. In addition, one-third of women treated for 5 years will eventually relapse within 15 years, as their tumors often become endocrine-resistant [4]. In luminal metastatic breast cancer, the objective response rate for tamoxifen treatment is only 30%, and 20% show stable disease. In regard to neo-adjuvant tamoxifen therapy, the clinical objective response rate is 30% to 60%, with 30% to 50% having stable disease, and about 3% having disease progression during treatment [5], [6].

Tamoxifen resistance may be primary or acquired. Insensitive molecular subtypes include luminal B, HER2+, and triple negative [7], [8]. With the exception of those subtypes, predictors of tamoxifen resistance are poorly defined, making it difficult to identify patients who are less likely to benefit from tamoxifen treatment. Some clinical and pathological factors that can predict early recurrence include cancers that are lymph node-positive or low ER-expressing as well as a multigene score called EndoPredict Index [3], [9]–[13]. The failure of tamoxifen to prevent many early relapses highlights the need for more effective therapies to improve clinical outcomes.

The ER signaling pathway plays a key role in the development of estrogen dominant breast cancer. However, this pathway is not the only survival pathway of tumors; thus, when the ER signaling pathway is blocked, the escape pathways function [14]. Importantly, these proliferative pathways can cross-talk with the ER pathway and regulate ER to affect endocrine therapy [15]–[18]. Activation of these pathways leads to the formation of ER-independent tumors. These pathways can be activated by amplification or overexpression of oncogenes, or by loss of function of downstream signaling molecules [19]. Studies have revealed that phosphorylation of protein kinase B (PKB/Akt) or mammalian target of rapamycin (mTOR) can directly alter sensitivity to tamoxifen, leading to tamoxifen resistance [20], [21]. However, the factors that induce ERα loss and activate Akt signaling remain unknown. Many clinical trials have been designed that block these escape pathways to increase the survival benefit in luminal breast cancer patients who have endocrine resistance.

Zinc finger 703 (ZNF703) is an oncogenic transcription factor that regulates many genes involved in multiple aspects of the cancer phenotype, including proliferation, increased self-renewal, and invasion [22]–[24]. ZNF703 was recently identified as a novel breast cancer oncogene in the 15% of breast cancers that harbor 8p12 amplifications, amplified second only to the well-known oncogenes, ERBB2 and cyclin D1 (CCND1) [25], [26]. Nevertheless, ZNF703 is still largely unknown in the breast cancer field; for example, it remains to be determined how the expression or function of the encoded protein can be regulated, and the major downstream effectors of its oncogenic functions have not been identified. In the present study, we hypothesized that ZNF703 might contribute to endocrine resistance. We assessed ZNF703 expression in clinical breast cancer specimens, and used breast cancer cell lines to investigate the role of and underlying mechanisms of ZNF703 in endocrine therapy.

## Results

### ZNF703 is Highly Expressed in Luminal Breast Tumors

**Figure 1:**
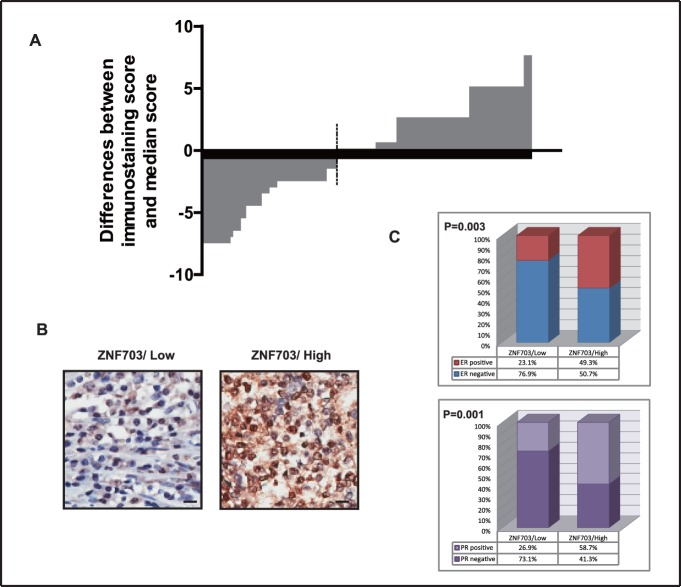
IHC staining of ZNF703 in 127 cases of breast cancer tissue microarrays. (A) Immunohistochemistry (IHC) scoring of ZNF703 in 127 breast cancer patients. The vertical axis indicates the difference between the score of each patient and the median score. The dotted line represents the cut-off point by which patients were divided into high and low ZNF703 expression groups. (B) Representative image of IHC staining for ZNF703 in breast cancer specimens. Left: low ZNF703 expression; Right: high ZNF703 expression. The bar represents 10 µm. (C) High ZNF703 expression was mainly present in ER+ and PR+ breast cancers. Left: proportion of patients with ER+ or ER− breast cancer in the high or low ZNF703 expression groups, respectively. Right: proportion of patients with PR+ or PR− breast cancer in the high or low ZNF703 expression groups, respectively. Also see Table S1.

We examined ZNF703 expression in breast cancer tissue microarray. In 127 breast cancer patients, the positive expression rate of ZNF703 protein was 91.3%. ZNF703 was mainly localized in the nucleus, although it was also expressed in the cytoplasm of some cells. All patients were divided into high and low ZNF703 expression groups (Figure 1A, B). In the high expression group, 49.3% of patients were ER+, whereas only 23.1% of the low expression group was ER+. This difference was statistically significant (Figure 1C, *P* = 0.003). Similarly, 58.7% of patients were PR+ in the high expression group compared to only 26.9% in the low expression group. This difference was also statistically significant (Figure 1C, *P* = 0.001). In addition, high ZNF703 expression was associated with an intermediate grade (Table S1, *P* = 0.04).

### Inhibitory Effects of TAM in Breast Cancer Cell Lines with High or Low ZNF703 Expression

**Figure 2:**
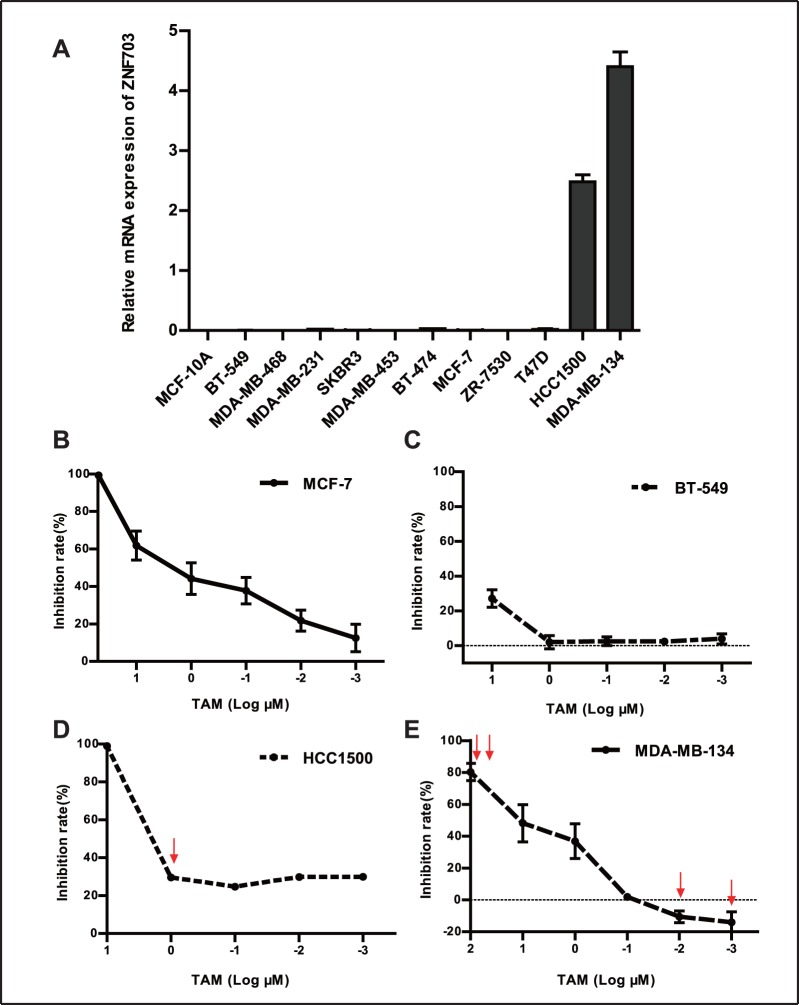
Breast cancer cell lines with high ZNF703 expression have different modes of tamoxifen resistance. (A) RT-PCR was used to evaluate ZNF703 mRNA expression levels in 11 breast cancer cell lines and the normal breast epithelial cell line MCF-10A. (B, C) MCF-7 and BT-549 cells were treated with DMSO or different concentrations of TAM, with the addition of 1 nM estradiol in each well. Inhibitory rates were examined using the SRB cell growth assay after 4 days. (D) Similar to MCF-7 cells, HCC1500 cells were treated with DMSO or different concentrations of TAM. The arrow shows where the inhibitory rate was less than 30% after treatment with 1 µM TAM. (E) MDA-MB-134 cells were treated with DMSO or different concentrations of TAM. The double-arrow shows where the inhibitory rate was 80% after treatment with 100 µM TAM. The single-arrow indicates that 10^−2^ µM or 10^−3^ µM TAM stimulated growth of MDA-MB-134 cells. Data are representative of three experiments and are presented as mean ± SD.

Given the involvement of ZNF703 in luminal-type breast cancer, we assessed ZNF703 mRNA expression levels in breast cancer cell lines by real time and reverse transcription polymerase chain reaction (RT-PCR) (Figure 2A). The MCF-7 luminal A-type breast cancer cell line had low ZNF703 expression levels, while MDA-MB-134 and HCC1500, luminal B-type breast cancer cell lines, had high expression levels, similar to observations in previous studies [22], [23]. The normal mammary epithelial cell line MCF-10A had almost no expression of ZNF703. Next, we performed experiments to determine if ZNF703 impacted 4-hydroxytamoxifen (TAM) sensitivity in breast cancer cell lines. The results showed that the inhibitory effects of TAM differed between breast cancer cell lines. In MCF-7 cells, increasing concentrations of TAM caused inhibitory rates to gradually increase (Figure 2B). At a concentration of 50 µM, the inhibitory rate reached 100%. In contrast, in the hormone receptor negative BT-549 cell line, the inhibitory rates were close to 0 within the range of 1 to 1×10^−3^ µM TAM, indicating that BT-549 cells were insensitive to tamoxifen (Figure 2C). Notably, although HCC1500 and MDA-MB-134 cell lines were much more sensitive to tamoxifen than BT-549, they exhibited particular modes of tamoxifen resistance. In HCC1500 cells, although the inhibitory rate reached 100% at 10 µM TAM, inhibitory rates of less than 30% were achieved in the range of 1 to 1×10^−3^ µM (Figure 2D). After treatment with 100 µM TAM, the inhibitory rate in MDA-MB-134 cells was still less than 100%. Notably, 10^−2^ µM and 10^−3^ µM tamoxifen promoted the growth of MDA-MB-134 cells (Figure 2E).

### ZNF703 Overexpression Activates the Akt Signaling Pathway and Reduces the Antitumor Effects of TAM

**Figure 3:**
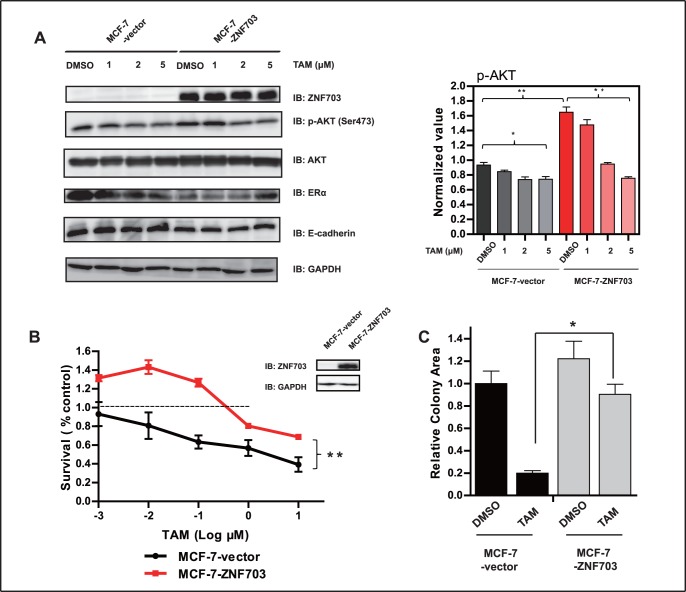
Overexpression of ZNF703 activates p-AKT and induces tamoxifen resistance. (A) MCF-7-vector and MCF-7-ZNF703 cells were treated with DMSO, 1 µM, 2 µM or 5 µM TAM for 24 hours, followed by western blot analysis of the indicated proteins. GAPDH was used as a loading control. ImageJ software was used to measure p-AKT levels and normalize to GAPDH levels (y-axis). (B) Western blot analysis of ZNF703 protein expression in MCF-7-vector and MCF-7-ZNF703 cells. MCF-7-vector and MCF-7-ZNF703 cells were treated with DMSO or different concentrations of TAM for 72 hours and inhibitory rates were examined. (C) Tamoxifen-mediated cell growth inhibition in MCF-7-vector and MCF-7-ZNF703 cells was evaluated by the colony formation assay. MCF-7-vector and MCF-7-ZNF703 cells were treated with DMSO or 1 µM TAM for 15 days, and colony forming areas were calculated to compare the differences before and after administration in both cell lines. Data are representative of three independent experiments and are presented as mean ± SD. *P<0.05, **P<0.01.

To determine the effect of ZNF703 on endocrine therapy, we overexpressed ZNF703 in MCF-7 cells (MCF-7-ZNF703 cells). These cells were treated with several concentrations of TAM, and the expression levels of several signaling proteins were measured. We detected considerable upregulation of phosphorylated Akt (p-Akt; *P*<0.01) and down-regulation of both ERα (*P*<0.05) and E-cadherin (*P*<0.05) in MCF-7-ZNF703 cells; total Akt protein levels remained unchanged (*P*>0.05). After treatment with increasing concentrations of TAM, p-Akt (Ser473) and ERα levels decreased in a dose-dependent manner in MCF-7-vector cells, whereas E-cadherin was upregulated (Figure 3A). In MCF-7-ZNF703 cells, increasing concentrations of TAM abrogated these effects, causing down-regulation of p-Akt (*P*<0.01) and upregulation of ERα (*P*<0.001).

**Figure 4:**
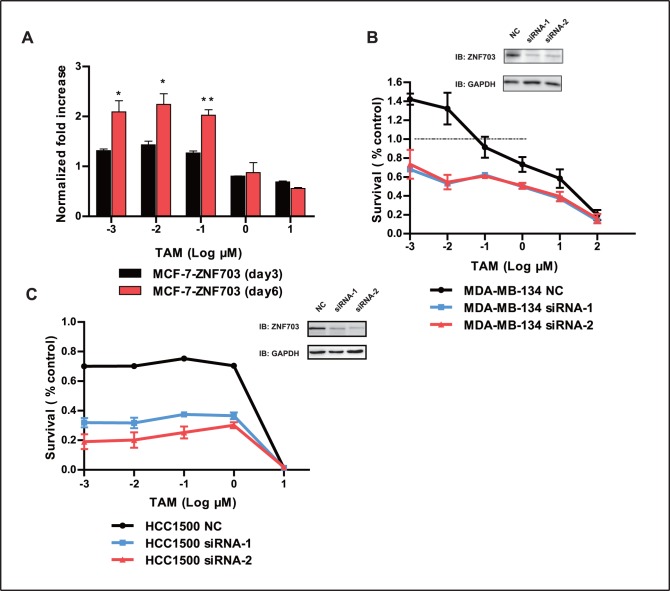
Increasing effects of TAM in MDA-MB-134 and HCC1500 cells after ZNF703 knockdown. (A) MCF-7-ZNF703 cells were treated with DMSO or different concentrations of TAM for 3 or 6 days, and the optical density (OD) was measured by the SRB assay. The y-axis represents the ratio of OD in TAM-treated cells compared to DMSO-treated cells. (B) Western blot analysis of ZNF703 and GAPDH in MDA-MB-134 non-targeting siRNA control (NC), MDA-MB-134 siRNA-1 or MDA-MB-134 siRNA-2 cells. Cells were treated with DMSO or different concentration of TAM for 6 days, and survival rates were examined. MDA-MB-134 NC vs. MDA-MB-134 siRNA-1 cells, *P*<0.05; MDA-MB-134 NC vs. MDA-MB-134 siRNA-2 cells, *P*<0.05; MDA-MB-134 siRNA-1 vs. MDA-MB-134 siRNA-2 cells, *P*>0.05. Statistical analyses were done by paired *t*-test. (C) Western blot analysis of ZNF703 and GAPDH in HCC1500 NC, HCC1500 siRNA-1, and HCC1500 siRNA-2 cells. Cells were treated with DMSO or different concentrations of TAM for 6 days, and survival rates were examined. HCC1500 NC vs. HCC1500 siRNA-1 cells, *P*<0.05; HCC1500 NC vs. HCC1500 siRNA-2 cells, *P*<0.05; HCC1500 siRNA-1 vs. HCC1500 siRNA-2 cells, *P*<0.05. Data is representative of at least three independent experiments and are presented as mean ± SD. *P<0.05, **P<0.01.

We next examined the effect of TAM on the survival of MCF-7-ZNF703 cells, and found that MCF-7-ZNF703 had significantly reduced inhibitory rates after treatment with various concentrations of TAM compared to MCF-7-vector cells (Figure 3B, *P*<0.01), indicating the occurrence of resistance. Surprisingly, low-dose TAM (10^−3^ to 10^−1^ µM) resulted in a 20% to 40% increase in the growth of MCF-7-ZNF703 cells, which is consistent with what we previously observed in MDA-MB-134 cells (Figure 2E). We performed a colony formation assay to verify the reduced inhibitory rate of MCF-7-ZNF703 cells upon treatment with TAM compared to MCF-7-vector cells (Figure 3C). We also observed that low-dose TAM (10^−3^ to 10^−1^ µM) stimulated the growth of MCF-7-ZNF703 cells in a time-dependent manner (Figure 4A), as 6 days of low-dose TAM treatment resulted in a 2-fold increase in cell growth compared to control cells.

### siRNA-mediated Depletion of ZNF703 Counteracts the Effects of Anti-TAM

To determine whether targeting ZNF703 could reverse tamoxifen resistance in ZNF703-overexpressing cell lines, small interfering RNAs (siRNAs) were used to knockdown ZNF703. siRNA depletion of ZNF703 in MDA-MB-134 cells significantly increased the inhibitory effect of TAM compared to control cells (Figure 4B, *P*<0.05). The stimulatory effect on cell growth by low-dose TAM was also eliminated. There was no difference between MDA-MB-134 siRNA-1 and MDA-MB-134 siRNA-2-treated cells (*P*>0.05).

Similar results were obtained in the HCC1500 cell line; siRNA depletion of ZNF703 revealed significantly increased inhibitory effects of TAM compared to control cells (Figure 4C, *P*<0.05). Treatment with siRNA-2 led to better knockdown efficiency of ZNF703 compared to treatment with siRNA-1; therefore, slightly lower survival rates were observed in HCC1500 cells treated with siRNA-2 (*P*<0.05).

### Rapamycin Increases the Effect of TAM on Cell Viability in ZNF703-overexpressing Cell Lines

**Figure 5:**
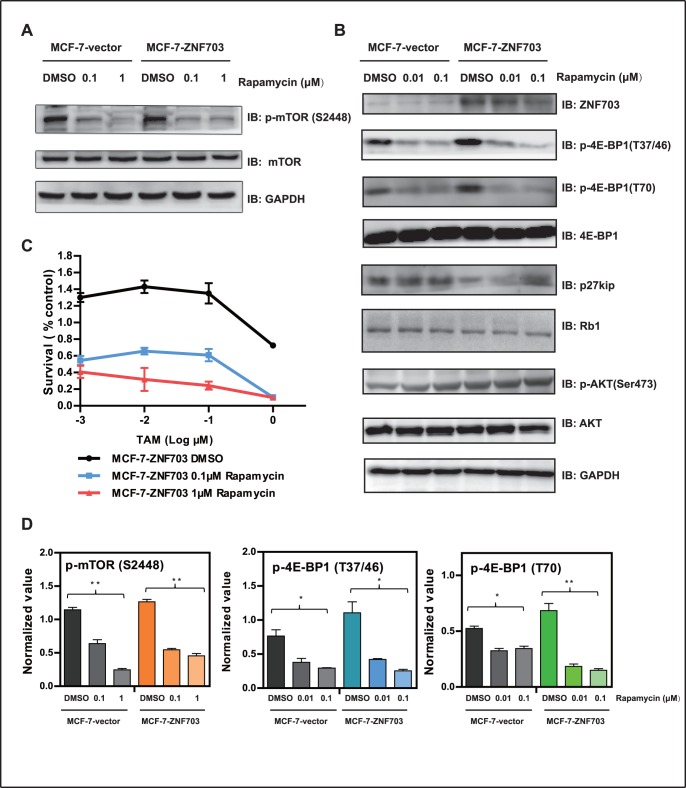
ZNF703-mediated activation of Akt/mTOR signaling can be inhibited by rapamycin. (A) MCF-7-vector and MCF-7-ZNF703 cells were treated with DMSO, 0.1 µM rapamycin, or 1 µM rapamycin for 24 hours before cell lysis. Western blot analysis of lysates using the indicated antibodies. A GAPDH antibody was used as a loading control (B) MCF-7-vector and MCF-7-ZNF703 cells were treated with DMSO, 0.01 µM rapamycin, or 0.1 µM rapamycin for 24 hours. Western blot analysis using the indicated antibodies. (C) DMSO or various concentrations of TAM were added to three MCF-7-ZNF703 cell groups that were treated with DMSO, 0.1 µM rapamycin, or 1 µM rapamycin for 72 hours. Survival rates were examined by the SRB assay. MCF-7-ZNF703 DMSO vs. MCF-7-ZNF703 0.1 µM rapamycin, *P*<0.001; MCF-7-ZNF703 DMSO vs. MCF-7-ZNF703 1 µM rapamycin, *P*<0.01; MCF-7-ZNF703 0.1 µM rapamycin vs. MCF-7-ZNF703 1 µM rapamycin, *P*>0.05, paired *t*-test was performed. (D) Expression of p-mTOR (S2448), p-4E-BP1 (T37/46) and p-4E-BP1 (T70) in (B) were measured using ImageJ software and normalized to GAPDH levels (y-axis). *P<0.05, **P<0.01.

To further elucidate whether the Akt signaling pathway was responsible for tamoxifen resistance in ZNF703-overexpressing cell lines, we treated cells with the mTOR inhibitor, rapamycin. Rapamycin significantly blocked p-mTOR (S2448) in MCF-7-vector cells in a dose dependent manner. p-mTOR (S2448) was upregulated in MCF-7-ZNF703 cells, and treatment with rapamycin significantly inhibited its expression (Figure 5A). In addition, rapamycin inhibited phosphorylation of eukaryotic translation initiation factor 4E-binding protein 1 (4E-BP1; T37/46 and T70), and increased phosphorylation of Akt in MCF-7 cells (Figure 5B), most likely due to negative feedback mechanisms [27]. We found that compared to MCF-7-vector cells, there was an increase in 4E-BP1 phosphorylation in MCF-7-ZNF703 cells; total 4E-BP1 protein levels remained unchanged. Levels of retinoblastoma protein 1 (RB1) and cyclin-dependent kinase inhibitor 1B (p27kip) decreased. Both p-4E-BP1 (T37/46) and p-4E-BP1 (T70) significantly decreased upon mTOR inhibition in both MCF-7-vector and MCF-7-ZNF703 cells, with subsequent recovery of p27kip expression (Figure 5B, D). In addition, rapamycin down-regulated ZNF703 expression.

To determine the effect of blocking mTOR on cell viability, we treated MCF-7-ZNF703 cells with either TAM alone or TAM in combination with rapamycin at a concentration selected based on its predetermined half maximal inhibitory concentration (IC50). TAM significantly decreased the number of viable cells when combined with rapamycin in MCF-7-ZNF703 cells (Figure 5C).

## Discussion

The ER signaling pathway plays a key role in the development of breast cancer. Tamoxifen has been widely used in the treatment of luminal breast cancer for over 30 years. However, nearly half of breast cancer patients are insensitive to tamoxifen therapy, some of whom relapse or have progressive disease when receiving tamoxifen, and thus have tamoxifen resistance. Although previous studies have reported various mechanisms of tamoxifen resistance, there are many “unknowns” that need further clarification.

ZNF703 is the most significant luminal potential oncogene [28]. ZNF703 is overexpressed in some breast cancer cell lines, but has low expression in normal mammary epithelial cells. Overexpression of ZNF703 increases genome instability and contributes to tumor aggressiveness. Moreover, one single-nucleotide polymorphism near ZNF703 is significantly associated with breast size [29]. IHC was used to evaluate ZNF703 expression in the breast cancer specimens, and high ZNF703 was mainly found in luminal breast cancers. In addition, overexpression of ZNF703 correlated with an intermediate grade, suggesting a moderate prognosis in breast cancer [30].

The modes of tamoxifen action were distinct between breast cancer cell lines with high or low ZNF703 expression. ZNF703 overexpression reduced the antitumor effect of tamoxifen. Both MDA-MB-134 and HCC1500 are luminal cell lines [31]–[33], with high ZNF703 levels. siRNA depletion of ZNF703 combined with tamoxifen increased its antitumor effects. Compared to luminal A-type, luminal B-type breast tumors are not sensitive to tamoxifen [7], [8]. Several studies use MDA-MB-134 and HCC1500 as representatives of luminal B-type breast cancer cell lines [22], [23], [34]. Our findings provide a basis for potential cellular mechanisms of tamoxifen resistance in luminal B breast cancer.

Preclinical experiments have shown that the phosphatidylinositol 3-kinase (PI3K)/Akt/mTOR pathway is a potential escape pathway that functions after the ER signaling pathway is blocked [20], [21]. In this regard, studies have shown that ER+ breast cancer patients with excessive activation of p-Akt (Ser473) who receive tamoxifen treatment have an increased recurrence rate and a reduced overall survival rate [35]. Levels of p-Akt (S473), p-mTOR (s2448), p-ER (S167), and p-ER (S305) expression can predict tamoxifen efficacy [36]. Early studies have shown that the mTOR signaling pathway is essential for the growth of estrogen-dependent luminal breast cancer cells. mTOR inhibitors with endocrine therapy synergizes to induce cell death [37]. Bolero-2 Phase III clinical trials showed that endocrine therapy combined with Everolimus can improve progression-free survival in luminal breast cancer patients with disease progression [20], [38], [39]. Overexpression of ZNF703 in MCF-7 cells can activate the Akt/mTOR signaling pathway. A mTOR inhibitor combined with siRNA knockdown of ZNF703 increased the antitumor effect of tamoxifen. Our results reveal that ZNF703 is a new molecule that can activate this pathway.

Previous studies have found that mutations or loss of ERα led to primary tamoxifen resistance. However, the factors that induce ERα loss remain unclear. Varying degrees of ERα loss can be detected in 15% to 20% of tamoxifen-resistant breast tumors [40]. ZNF703 overexpression decreased ERα levels, leading to less tamoxifen targets, and high-dose TAM resulted in the recovery of ERα. The phenomenon that ZNF703 repressed ERα expression is not contradictory with our results showing that ZNF703 was highly expressed in luminal breast cancer. However, further studies are needed to verify if ZNF703 is negatively correlated with ER expression in breast cancer patients.

The ER signaling pathway is closely related to the growth factor receptor pathway. First, the ER signaling pathway can be modulated by membrane receptor tyrosine kinases (RTKs), such as epidermal growth factor receptor (EGFR), HER-2, and insulin-like growth factor-1 receptor (IGF1-R) [15]. These RTKs can eventually phosphorylate ER and its co-activators or co-repressors, thereby affecting their function [16], [41], [42]. Second, estrogen increases the expression of growth factors, such as transforming growth factor (TGF)-α and IGF1, and activates downstream signaling [43]. In turn, activated PI3K/Akt and p42/44 mitogen-activated protein kinase (MAPK) pathways can reduce the expression of ER and PR [44]–[49]. In addition, ER which functions in non-nuclear transcription, can alter the amount of proteins regulated by growth factor receptors [17], [18]. Excessive activation of growth factor receptor pathways can increase the number of non-nuclear ERs and non-transcriptional activity, while non-nuclear ER activity can also be activated by estrogen or tamoxifen, resulting in tamoxifen resistance. Overexpression of RTKs causes tamoxifen to increase the function of non-nuclear ER and activate RTK downstream pathways, leading to tamoxifen resistance. We found that fibroblast growth factor receptor 4 was upregulated in MCF-7-ZNF703 cells (data not shown). Interestingly, mimicking endogenous estrogen levels, low-dose tamoxifen stimulated the growth of tumor cells upon ZNF703 overexpression. Therefore, ZNF703 is likely to increase the expression of RTKs as well as non-nuclear ER activity, which further activates the PI3K/Akt pathway, leading to cell proliferation and antagonism of tamoxifen.

Many regulatory proteins related to the cell cycle have been found. For example, cyclin-dependent kinase inhibitors (CDKI) negatively regulate the cell cycle, and RB is a negative regulator of the transition from the G1 to S phase. Studies have shown that low p27 [50]–[52], CCND1 expression abnormalities [53], [54], and RB inactivation are associated with tamoxifen resistance [55]. We found that overexpression of ZNF703 caused down-regulation of p27 and RB1, as well as upregulation of CCND1 (data not shown). These results suggest that ZNF703 can participate in tamoxifen resistance through intrinsic cell cycle mechanisms.

Furthermore, fibroblast growth factor receptor 1 amplification (also in *8p12*) leads to tamoxifen resistance [56]. Several signaling molecules, including the Hedgehog pathway molecules [57], ΔEF1 [58], CUEDC2 [59], LMTK3 [60], [61], HOXB7 [62], and HER-1/HER-2 [63] are associated with tamoxifen resistance. However, more research is needed to determine which of these proteins drive tamoxifen resistance.

In summary, our data demonstrates the important role of ZNF703 as a novel molecule in tamoxifen resistance induced by activation of the Akt/mTOR signaling pathway and down-regulation of ERα. Our findings provide a potential mechanism of early- or late-recurring tamoxifen-resistant luminal breast cancers. It can be predicted that targeting ZNF703 and its downstream signaling pathway can increase the efficacy of endocrine therapy in patients with ZNF703-overexpressing luminal breast cancer. In addition, tamoxifen should be used with caution in patients with ZNF703 overexpression.

## Materials and Methods

### Ethics Statement

Written informed consent was obtained from all patients. The study protocol was designed according to the principles of the Helsinki guidelines, and approved by the Institutional Review Board of Ruijin hospital (China).

### Antibodies, Hormones, and Reagents

Anti-ZNF703 polycolonal antibody was used at a 1∶50 dilution for immunohistochemistry (Sigma, St. Louis, MO, USA). The following monoclonal antibodies were used for western blotting: p27kip at a 1∶500 dilution (Abcam, Cambridge, England), GADPH at a 1∶10,000 dilution (Cell Signaling Technology, Boston, MA, USA), ZNF703 (Abcam), ERα, RB1, AKT, p-AKT (Ser473), p-mTOR, mTOR, 4E-BP1, p-4E-BP1(T37/46), p-4E-BP1 (T70), and E-cadherin (all at a 1∶1000 dilution purchased from Cell Signaling). 17-β-oestradiol (E2) and TAM were purchased from Sigma, and rapamycin was purchased from Selleck Chemicals (Houston, TX, USA).

### Cell Culture

All breast cancer cell lines were purchased from American Type Culture Collection (Manassas, VA, USA) and cultured in complete growth medium as described by the company. Cell lines were monitored by performing polymerase chain reaction (PCR) using the Mycoplasma Detection Kit (Invitrogen, Carlsbad, CA, USA) on the Applied Biosystems Real-Time PCR System. Briefly, MDA-MB-134 cells were grown in L-15 medium (Gibco, Carlsbad, CA, USA) with 20% fetal bovine serum (FBS; Gibco); HCC1500 and BT-549 cells were cultured in RPMI 1640 medium (Gibco) supplemented with 2 mM L-glutamine with 10% FBS; MCF-7 cells were cultured in Minimum Essential Media (Gibco) supplemented with 10% FBS.

### ZNF703 Stable Cell Lines

The pCMV6–myc-DDK and pCMV6–myc-DDK-ZNF703 plasmids were purchased from Origene Company. For stable transfections, MCF-7 cells were transfected with pCMV6–Myc-DDK or pCMV6–Myc-DDK-ZNF703 plasmids with Lipofectamine LTX (Invitrogen) according to the manufacturer’s instructions. Pools of stable polyclonal cells were established under continuous selection with geneticin (G418, Invitrogen) for 7 days.

### Immunoblot Analysis

The indicated cell lines were grown in 6-well plates, treated as indicated, and lysed in lysis buffer (20 mM Tris-HCl (pH 7.4), 150 mM NaCl, 0.1% SDS, 1% Triton X-100, 1% sodium deoxycholate) containing protease and phosphatase inhibitors (Beyotime, Shanghai, China). Lysates were subjected to SDS-PAGE and western blotting with the indicated antibodies as previously described [62]. Briefly, cell lysates were resolved by SDS-PAGE and electrotransferred to polyvinylidene difluoride membranes (Millipore, Billerica, MA, USA). After blocking at room temperature in 5% nonfat milk in TBST (20 mM Tris-HCl (pH 7.4), 150 mM NaCl, and 0.1% (w/v) Tween 20) buffer for 1 h, the membranes were incubated overnight at 4°C with the appropriate primary antibodies. The next day, the membranes were washed three times with TBST, and then incubated with peroxidase-conjugated secondary antibodies for 1 h at room temperature. After three more washes with TBST, proteins were visualized using a Chemiluminescence Detection Kit (Millipore). GAPDH was used as a loading control.

### Quantitative RT-PCR

Quantitative real-time PCR was performed using SYBR (Takara, Japan) on the ABI Prism 7500 fast system (Applied Biosystems, Carlsbad, CA, USA) using standard curve method. The following primers were used: ZNF703 primer: 5′-ACCCTCTTCCAGACACCTAAGC-3′ (forward); 5′-TTGAGGAAAGGCATTAAACTCG-3′ (reverse); ERα primer: 5′-AAGAAAGAACAACATCAGCAGTAAAGTC-3′ (forward); 5′-GGGCTATGGCTTGGTTAAACAT-3′(reverse). GAPDH primer: 5′-CTTAGCACCCCTGGCCAAG-3′ (forward); 5′-GATGTTCTGGAGAGCCCCG-3′ (reverse).

### siRNA Transfection and Cell Line Drug Sensitivity

Target sequences for ZNF703 siRNA using RNAiMAX (30 nM final concentration, Invitrogen) were as follows: siRNA-1 (sense strand- 5′ CCACACACUUUGGGCCUAA dTdT 3′; antisense strand- 3′ dTdT GGUGUGUGAAACCCGGAUU 5′), siRNA-2 (sense strand- 5′ GGACCCUACUAUUCGCCAU dTdT 3′; antisense strand- 3′ dTdT CCUGGGAUGAUAAGCGGUA 5′). Non-targeting control (NC) siRNA was designed and synthesized by Guangzhou RuiBoBio (Guangzhou, China). For assessment of endocrine therapy, cells were maintained in phenol red-free media, supplemented with 10% charcoal/DCC stripped serum (Tissue Culture Biologicals, Long Beach, CA, USA) and 1 nM estradiol, and then plated in 96-well plates and treated for 3 to 6 days with a range of TAM concentrations. Cell proliferation was determined using the sulforhodamine B (SRB) (Sigma) assay. Relative growth was calculated as the value relative to dimethyl sulfoxide (DMSO) treated cells (control).

### Colony-forming Assay

Cells were seeded into 6-well plates (1000 cells per well). Standard growth medium, with TAM or DMSO, was changed every other day. On day 15, colonies were fixed in 10% methanol, 10% acetic acid, and 80% ddH_2_O, and then stained with crystal violet (0.5% w/v).

### Analysis of ZNF703 Expression in Tissue Microarray

Tissue specimens were obtained from archived tissue samples of patients with primary breast cancer who underwent surgical treatment at Ruijin Hospital (China) between January 2001 and December 2003. The selection criteria were as follows: (1) subjects had a diagnosis of stage I-III primary breast cancer and no history of other tumors; (2) subjects had complete clinical data, such as age, gender, stage, lymph node status, tumor size (as according to pathology reports), and pathological type. Patients receiving chemotherapy or radiotherapy prior to surgery were excluded. IHC analysis of ZNF703 protein was performed on tissue microarray sections with antibodies against ZNF703, as previously described [64]. ZNF703 expression was independently evaluated by two pathologists, both of whom were blinded to the clinicopathologic data. Specimens were assigned scores according to the intensity of nuclear staining (no staining = 0; weak staining = 1, moderate staining = 2, strong staining = 3), and to the percentage of stained cells (0% = 0, 1–20% = 1, 21–40% = 2, 41–60% = 3, 61–80% = 4, 81–100% = 5). The final immunoreactive score was determined by multiplying the intensity score by the score representing the percentage of stained cells, and ranged from 0 (minimum score) to 15 (maximum score). Cases were further classified into negative/low and high expression groups according to the median score of nucleic acid staining. Evaluation of hormone receptor expression was based on the Allred scoring method [65].

### Statistics

Each experiment was repeated at least three times. Data were presented as mean ± the standard deviation (SD) of each experiment. Data analysis was performed using SPSS 17.0. Student’s *t*-test was used to evaluate the numerical data. Chi-square test was used for comparisons of categorical data. Statistical tests were two-sided, and P values less than 0.05 were considered statistically significant.

## Supporting Information

Table S1 **ZNF703 expression in 127 breast cancer patients.**
(DOC)
