# Supplementary material for: Luminal Breast Cancer Cell Lines Overexpressing ZNF703 Are Resistant to Tamoxifen through Activation of Akt/mTOR Signaling
Source: PLoS One. 2013 Aug 26;8(8):e72053. doi: 10.1371/journal.pone.0072053 (PMC3753350; doi:10.1371/journal.pone.0072053)
Supplement: Table S1 — ZNF703 expression in 127 breast cancer patients. (DOC) [file pone.0072053.s001.doc]

**Table S1.** ZNF703 expression in 127 breast cancer patients

| **Characteristics** | **Total**  **No.** | **Negative/Low**  **No(%)** | **High**  **No(%)** | **P value ^b^** |
| --- | --- | --- | --- | --- |
| **Age** | 122 ^a^ |  |  | 0.78 |
| <=50 |  | 20 (42.6) | 30 (40.0) |  |
| >50 |  | 27 (57.4) | 45 (60.0) |  |
| **ER status** | 127 |  |  | 0.003 ^b^ |
| Negative |  | 40 (76.9) | 38 (50.7) |  |
| Positive |  | 12 (23.1) | 37 (49.3) |  |
| **PR status** | 127 |  |  | 0.001 ^b^ |
| Negative |  | 38 (73.1) | 31 (41.3) |  |
| Positive |  | 14 (26.9) | 44 (58.7) |  |
| **Lymph node metastases** | 86 ^a^ |  |  | 0.35 |
| No |  | 19 (61.3) | 28 (50.9) |  |
| Yes |  | 12 (38.7) | 27 (49.1) |  |
| **Grade** | 116 ^a^ |  |  | 0.04 ^b^ |
| 1 |  | 4 (8.3) | 15 (22.1) |  |
| 2 |  | 21 (43.8) | 34 (50.0) |  |
| 3 |  | 23 (47.9) | 19 (27.9) |  |
| **Pathological type** | 127 |  |  | 0.22 |
| IDC |  | 47 (90.4) | 62 (82.7) |  |
| other |  | 5 (9.6) | 13 (17.3) |  |

^a^ Missing data not calculated statistically. ^b^ P values less than 0.05 considered statistically significant.
